# Identification of Selective α-Glucosidase Inhibitors via Virtual Screening with Machine Learning

**DOI:** 10.3390/molecules30193996

**Published:** 2025-10-06

**Authors:** Fengyu Guo, Jiali Shi, Wenhua Jin, Feng Zhang, Hao Chen, Weibo Zhang, Yan Zhang, Chen Chong, Fazheng Ren, Pengjie Wang, Ping Liu

**Affiliations:** 1 Department of Nutrition and Health, China Agricultural University, Beijing 100083, China; b20213060484@cau.edu.cn (F.G.); sjl1998@cau.edu.cn (J.S.); jinwenhua@cau.edu.cn (W.J.); zhangweibo@cau.edu.cn (W.Z.); chenchong409@cau.edu.cn (C.C.); renfazheng@cau.edu.cn (F.R.); 2 National Technology Innovation Center for Dairy, Huhehaote 010000, China; wangjingtyry@163.com; 3 Suzhou Institute of Systems Medicine, Chinese Academy of Medical Sciences & Peking Union Medical College, Suzhou 215123, China; ch@ism.cams.cn; 4 College of Food Science and Engineering, Gansu Agricultural University, Lanzhou 730070, China; zhangyan@gsau.edu.cn

**Keywords:** α-glucosidase selective inhibitors, machine learning, virtual screening

## Abstract

Given the limitations of clinical and potent natural α-glucosidase inhibitors, novel selective inhibitors are urgently needed. To accelerate discovery, we employed machine learning-integrated virtual screening to rapidly evaluate a library of 100 K^+^ compounds, identifying a series of selective α-glucosidase inhibitors. Activity validation demonstrated that these inhibitors exhibit significantly enhanced selectivity and potency compared to the positive control acarbose. Mechanistic studies through inhibition kinetics and fluorescence quenching revealed their improved inhibitory profile. Molecular docking indicates that key interactions—hydrogen bonding or salt bridges with the catalytic residue ASP526—strengthen binding within the active site. These interactions competitively obstruct enzyme-substrate binding, thereby amplifying inhibition. In vitro and in vivo starch digestion assays further corroborated these findings.

## 1. Introduction

In the treatment of obesity and diabetes, postprandial glycemic control has gained significant attention due to its close association with diabetic complications [1]. Postprandial glycemic regulation primarily involves the inhibition of starch-digesting enzymes, particularly salivary amylase and α-glucosidase, which are secreted by the pancreas [2,3]. Currently, widely used clinical drugs such as acarbose, voglibose, and miglitol predominantly inhibit both α-amylase and α-glucosidase [4,5]. However, the extensive inhibition of these enzymes can result in incomplete starch digestion, leading to the accumulation of undigested starch. This, in turn, may cause various gastrointestinal side effects, including intestinal flatulence, as undigested starch undergoes microbial fermentation in the colon [6]. Therefore, to improve the clinical efficacy and patient experience with starch-digesting enzyme inhibitors, the development of new compounds must overcome the significant drawbacks of current therapeutic agents [7]. Numerous compounds with selective inhibitory activity against α-glucosidase, such as apigenin and quercetin, have been reported, with most derived from the isolation of natural products. However, these compounds often exhibit weak selective inhibition of α-glucosidase [8,9]. Therefore, there is a need to explore a broader chemical space in search of more potent and selective α-glucosidase inhibitors.

Various technologies have been developed to screen molecules across large chemical spaces, including high-throughput screening of compound libraries, combinatorial chemistry, and computer-aided drug design [8,9]. With significant advances in structural biology and the availability of high-performance computing resources, virtual screening has emerged as a powerful alternative to laboratory high-throughput screening. It offers clear advantages in terms of efficiency, cost-effectiveness, flexibility, and hit rate, making it one of the most widely utilized methods in drug discovery [10]. However, the application of computer simulation screening to large-scale compound libraries is limited by the substantial computational resources required, which can hinder its broader use [11]. In recent years, the integration of multiple computational methods into in silico screening has proven effective in conserving computational resources and expanding its scope of application [11,12].

**Figure 1:**
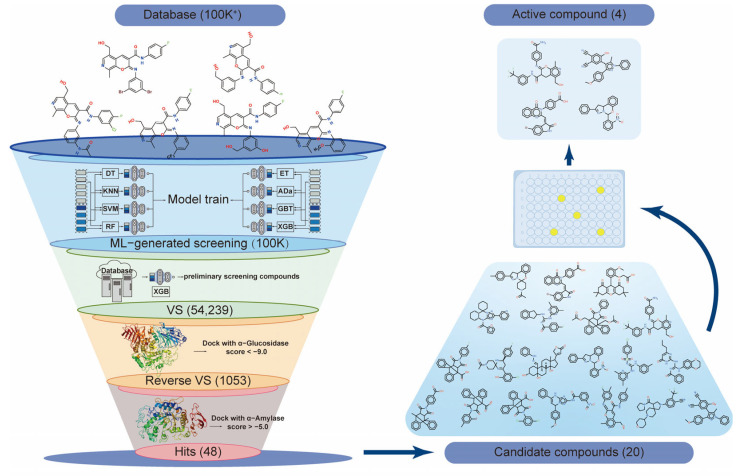
Flowchart of the screening strategy for selective α-glucosidase inhibitors using machine learning integrated with virtual screening. ML, machine learning; VS, virtual screening.

In this study, we employed a combination of machine learning (ML) and virtual screening (VS) technology (Figure 1) to identify α-glucosidase-selective inhibitors from a large compound library containing over 100 K compounds. To identify promising candidates, we investigated the structural characteristics, selective inhibitory mechanisms, and in vivo effects on blood sugar regulation. As a result, four potential α-glucosidase-selective inhibitors were discovered.

## 2. Results and Discussion

### 2.1. Construction of the Machine Learning Model

Performing variable selection before modeling is essential to address covariance issues arising from dataset complexity, using methods such as forward/backward/stepwise screening, optimal subset selection, genetic algorithms, variable clustering, or PCA. Screening reduces model complexity and redundancy, enhancing robustness and adaptability [13]. To reduce the dimensionality of independent variables and remove unnecessary variables, we first clustered the chemical dataset. The 1562 RDKit molecular descriptors underwent variable reduction via clustering and principal component analysis, selecting the first 30 clustered variables. Within each cluster, the variable with the smallest 1 − R^2^ ratio was chosen as the representative. The full set of 1562 descriptors (training/validation sets) and the 30 selected variables are provided in Supplementary Data Table S1 and Table S2, respectively. Active (1) and inactive (0) compound labels served as dependent variables to construct models.

To further select the optimal parameters for the model, grid search and 10-fold cross-validation were used. When the average accuracy of the model reached its maximum value, the corresponding model parameters were considered the optimal parameters for modeling. This method avoids overfitting or randomness that may occur due to a single random division. For DT, max_depth is set to 10 and n_estimators to 300, with all other parameters at default. For KNN, n_neighbors is optimized to 5, the weight is set to distance, and other parameters remain default. In SVM, ε controls training error, and the model uses a radial basis function (kernel = “rbf”). Cross-validation is used to select the optimal penalty coefficient C, set to 1 for this study to balance model complexity and error. For RF, n_estimators is optimized to 200 with max_depth = 3, while other parameters are default. Similarly, for ERT, n_estimators is set to 200 and max_depth to 3. In AdaBoost, n_estimators and max_depth are optimized to 200 and 5. Both GBT and XGBoost have optimized n_estimators and max_depth set to 200 and 5, respectively. Through the above parameter optimization process, a balance between complexity and generalization ability was ensured for each model, thereby providing a reliable model foundation for subsequent research.

### 2.2. Validation of the Machine Learning Model

**Figure 2:**
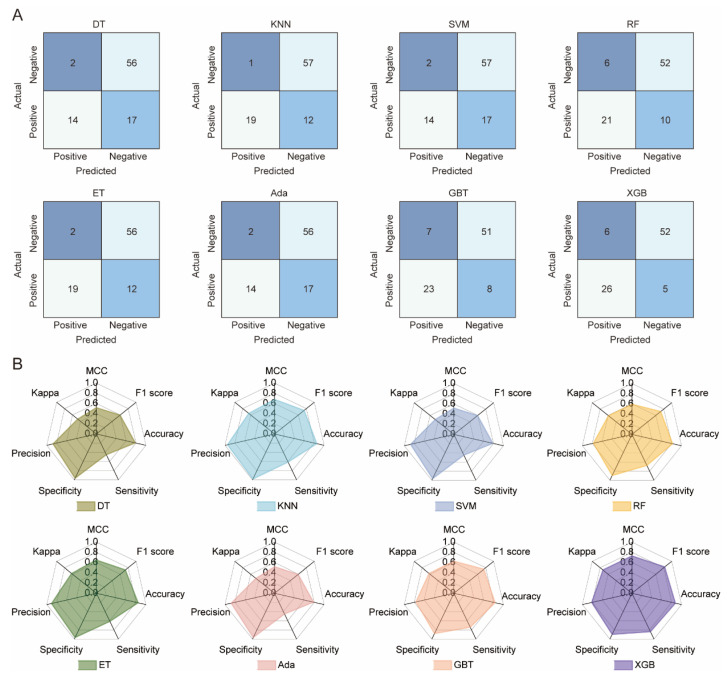
Performance evaluation of eight machine learning models on the test set. (**A**) Comparative confusion matrices for classification results. (**B**) Radar chart illustrating comprehensive model performance metrics (e.g., accuracy, precision, recall, F1-score, AUC). DT, decision tree; kNN, k-nearest neighbors; SVM, support vector machine; RF, random forest; ERT, extremely randomized trees; AdaBoost, adaptive boosting; GBT, gradient boosted trees; and XGBoost, eXtreme gradient boosting.

**Figure 3:**
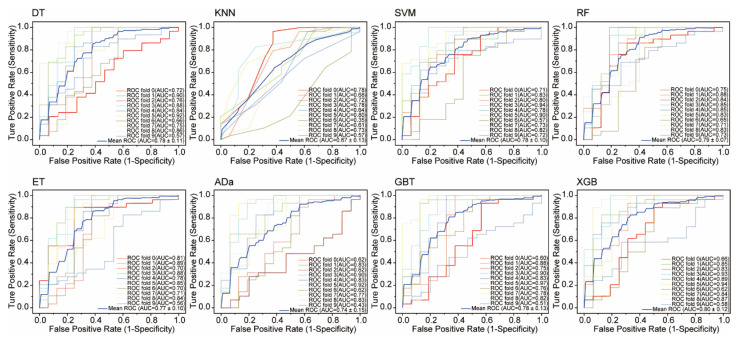
Ten-fold cross-validation performance of eight machine learning models. DT, decision tree; kNN, k-nearest neighbors; SVM, support vector machine; RF, random forest; ERT, extremely randomized trees; AdaBoost, adaptive boosting; GBT, gradient boosted trees; and XGBoost, eXtreme gradient boosting.

The statistical results for the eight ML models on the test set are shown in Figure 2. DT and SVM have the worst performance, with the lowest TP (14) and various other suboptimal values (Accuracy: 0.787, Sensitivity: 0.452, etc.). KNN, RF, ET, Ada, and GBT show similar TP values (19–23). XGB outperforms all models, achieving the highest values for Accuracy (0.876), Sensitivity (0.839), Kappa (0.730), MCC (0.730), and F1 score (0.825), while KNN leads in Precision (0.983) and Specificity (0.950). The TOPSIS algorithm was used to evaluate each model, considering seven parameters. Results show XGB is the best model, followed by KNN and GBT. Overall, XGB outperforms the others on the randomized test set, demonstrating its superior predictive ability. To avoid overfitting, 10-fold cross-validation was used to evaluate XGB and other models. Figure 3 displays the ROC curves for all models. XGB shows the highest average area under the curve (AUC) at 0.80, followed by DT, RF, SVM, and GBT with AUCs of 0.78, 0.79, 0.78, and 0.78, respectively. The kNN model has the lowest average accuracy at 0.67.

Above all, the XGB model demonstrates strong capability in recognizing both active and inactive α-glucosidase targets. Therefore, it was employed in the initial step of ligand-based drug screening. The model classifies the database and assigns an activity probability to each compound, which serves as the selection criterion. Predicted results are output as active molecules (labeled: 1) or inactive molecules (labeled: 0) based on the XGB model’s probability. The results revealed that 54,239 active compounds were identified from the 100 K^+^ compound library (Supplementary Data Table S6).

### 2.3. Virtual Screening of a Machine Learning-Generated Compound Library

Molecular docking is a structure-based VS method that simulates the binding of small molecules to proteins in their three-dimensional conformations. To identify active compounds that effectively bind to proteins, molecular docking between α-glucosidase (2QMJ) and 54,239 potentially active compounds from XGB screening was performed as the second step. Compounds with docking scores lower than −8.5 were selected as potential α-glucosidase inhibitors, resulting in 1053 compounds. These were then docked with α-amylase (1XCW), and compounds with scores higher than −5.0 were excluded, leaving 48 potential α-glucosidase selective inhibitors (Supplementary Table S1). Following structural and clustering analyses, 20 representative compounds were selected for preliminary activity validation (Supplementary Table S2).

**Figure 4:**
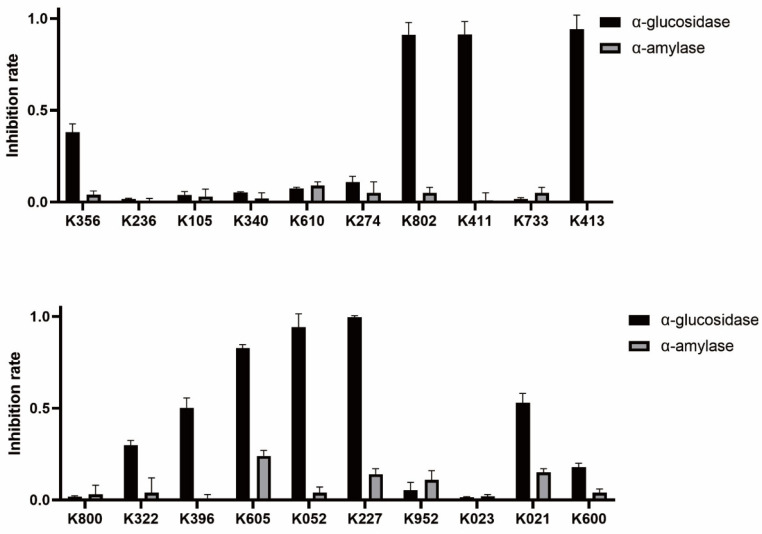
Inhibitory effects of candidate compounds on α-glucosidase and α-amylase at a concentration of 250 µM.

Among the 20 clustered compounds, four (K802, K411, K413, and K052) exhibited nearly 100% inhibition of α-glucosidase at a concentration of 250 µM, with minimal inhibition of α-amylase (Figure 4). This selective inhibition profile is consistent with the characteristics of potential α-glucosidase-selective inhibitors, supporting their potential for further activity evaluation.

### 2.4. Affinity Determination Between Selected Compounds and α-Glucosidase

To evaluate the selective inhibitory activity of the four candidate compounds, their IC_50_ values were determined, as presented in Table 1. In this study, the IC_50_ values of acarbose against α-glucosidase and α-amylase were 562.22 ± 20.74 µM and 228.23 ± 22.34 µM, respectively. These results suggest that acarbose exhibits a potent inhibitory effect on α-amylase and a comparatively weaker effect on α-glucosidase.

**Figure 5:**
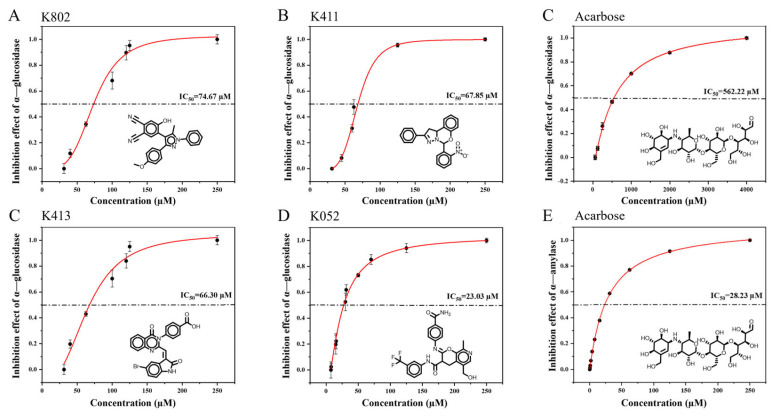
IC_50_ determination of candidate compounds. (**A**–**E**) Inhibitory activity of K802, K411, acarbose, K413, and K052 against α-glucosidase, respectively. (**F**) Inhibitory activity of acarbose against α-amylase.

Compared with acarbose, the screened compounds exhibited markedly weaker inhibitory effects on α-amylase, while showing significantly enhanced inhibition of α-glucosidase. Among them, K052 demonstrated the strongest α-glucosidase inhibition, with an IC_50_ value of 23.03 ± 3.27 μM (Figure 5). This alteration in inhibitory selectivity satisfies the criteria for a selective α-glucosidase inhibitor, offering the potential to control postprandial blood glucose levels while minimizing side effects associated with α-amylase inhibition.

Lim et al. (2022) reported that the flavonoid 3′,4′-dihydroxyflavonol exhibited selective inhibition, with IC_50_ values of 118.65 μM for α-amylase and 34.31 μM for α-glucosidase [14]. Moreover, Sun et al. (2017) showed that chalconaringenin, a chalcone analog, had an IC_50_ > 100 μM for α-amylase and 20.02 μM for α-glucosidase [15]. K052 identified in this study demonstrates superior selectivity and inhibitory potency against α-glucosidase compared to these reported natural selective inhibitors.

### 2.5. Inhibition Kinetics of Potential α-Glucosidase Selective Inhibitors

**Figure 6:**
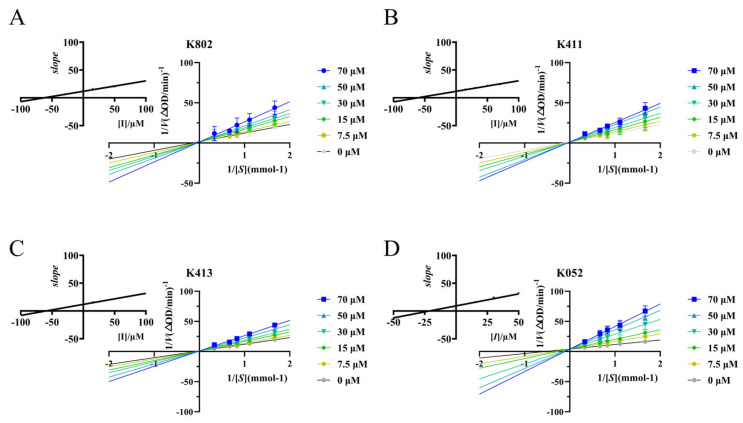
Kinetic curves of α-glucosidase inhibition by different inhibitors. (**A**) Lineweaver–Burk plots show the enzyme kinetic curves of alpha-glucosidase at different K802 inhibitor concentrations. The inset displays a quadratic curve of the slope versus K802 inhibitor concentration. (**B**) Lineweaver–Burk plot displays the enzyme kinetic curves of α-glucosidase at different K411 inhibitor concentrations. The inset shows the quadratic curve of slope versus K411 inhibitor concentration. (**C**) Lineweaver–Burk plot displays the enzyme kinetic curves of α-glucosidase at different K413 inhibitor concentrations, with the inset showing a quadratic plot of slope versus K413 inhibitor concentration. (**D**) Lineweaver–Burk plot displays the enzyme kinetic curves of α-glucosidase at different K052 inhibitor concentrations, with the inset showing a quadratic plot of slope versus K052 inhibitor concentration.

Due to the influence of assay conditions on IC_50_ values, cross-study comparisons of inhibitory activity require detailed kinetic analysis for accurate interpretation. Using Lineweaver-Burk plots, we determined the inhibition type, maximum initial reaction rate (*V_max_*), and Michaelis constant (*K_m_*). As shown in Figure 6, all lines intersect on the *y*-axis. Increasing inhibitor concentration decreased the x-intercept and increased the slope, while the y-intercept remained constant. This pattern indicates an unchanged *V_max_* and a decreased *K*_m_, characteristic of competitive inhibition [16]. Further kinetic analysis (Table S3) confirmed that the screened compounds act as competitive inhibitors of α-glucosidase [17]. Moreover, all four compounds exhibited markedly stronger binding affinity to α-glucosidase than acarbose, as evidenced by substantially lower *K_ic_* values. These results align with the IC_50_ determinations and further substantiate the superior inhibitory potential of the screened compounds.

### 2.6. Fluorescence-Based Analysis of Binding Mechanism

To further examine the inhibitor-protein interaction at the molecular level, we explored a fluorescence quenching assay. α-Glucosidase contains intrinsic fluorophores, primarily tryptophan (Trp) and tyrosine (Tyr) residues, which enable its fluorescence at specific excitation wavelengths. Notably, fluorescence intensity directly correlates with enzyme concentration in solution [18]. When inhibitors bind to key residues within the enzyme’s active site (e.g., Trp406, Tyr214) or their proximity, fluorescence is quenched. This attenuation occurs because the inhibitor alters the local environment of the fluorophore, reducing its fluorescence. Thus, the inhibitor must bind to or near these specific sites.

**Figure 7:**
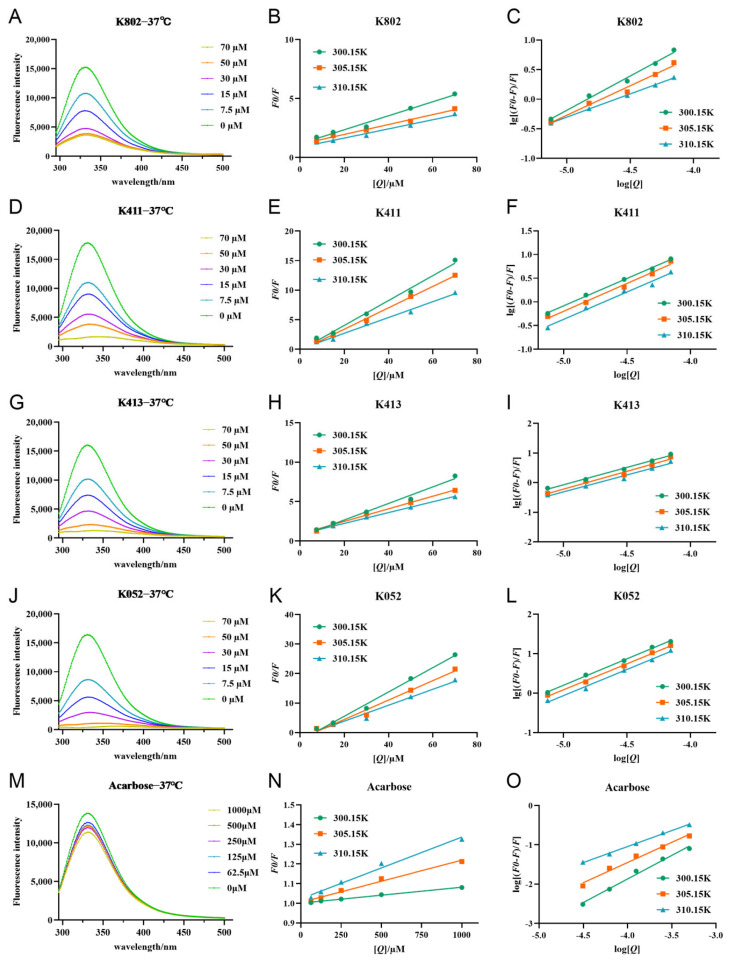
Fluorescence quenching profiles and Stern–Volmer plots of each inhibitor with α-glucosidase. (**A**,**D**,**G**,**J**,**M**) are the fluorescence quenching patterns of α-glucosidase by compounds. (**B**,**E**,**H**,**K**,**N**) are the fitting curves of *F*_0_*/F* and substrate concentration [*Q*] of α-glucosidase for compounds. (**C**,**F**,**I**,**L**,**O**) is the fitting curve of lg[(*F*_0_*/F*)/*F*] and substrate concentration log[*Q*] for α-glucosidase of compounds.

Fluorescence quenching can be classified into static quenching, dynamic quenching, or a combination of both. In this study, Stern-Volmer parameters at three different temperatures (300.15 K, 305.15 K, and 310.15 K) were used to investigate the type of fluorescence quenching caused by the inhibitor on α-glucosidase. Interestingly, the addition of the inhibitor caused a redshift in the enzyme’s maximum emission wavelength (λem) in some fluorescence spectra, suggesting potential partial structural unfolding of α-glucosidase upon inhibitor binding [18]. The interaction between the inhibitors and α-glucosidase likely involves microenvironmental changes in the local spatial structure and backbone conformation of the protein, induced by aromatic heterocyclic and hydrophobic groups of Trp and Tyr residues. The λem redshift further supports this potential structural unfolding [19]. As shown in Figure 7, the fluorescence intensity increases linearly with inhibitor concentration, indicating a single fluorescence quenching mechanism for α-glucosidase. Additionally, the *K_q_* value exceeds the maximum scattering collisional burst constant (2.0 × 10^10^ L/mol-s, Table S3), suggesting that the fluorescence quenching mechanism is primarily static rather than dynamic [20]. Since the *K_sv_* value reflects the binding interaction between the inhibitor and α-glucosidase, higher *K_sv_* values indicate stronger binding. Interestingly, the *K_sv_* values showed a trend of K052 > K413 > K411 > K802 > acarbose (Table S3), suggesting that the four compounds obtained from the screening exhibit higher binding affinity, which is consistent with previous experimental results.

**Figure 8:**
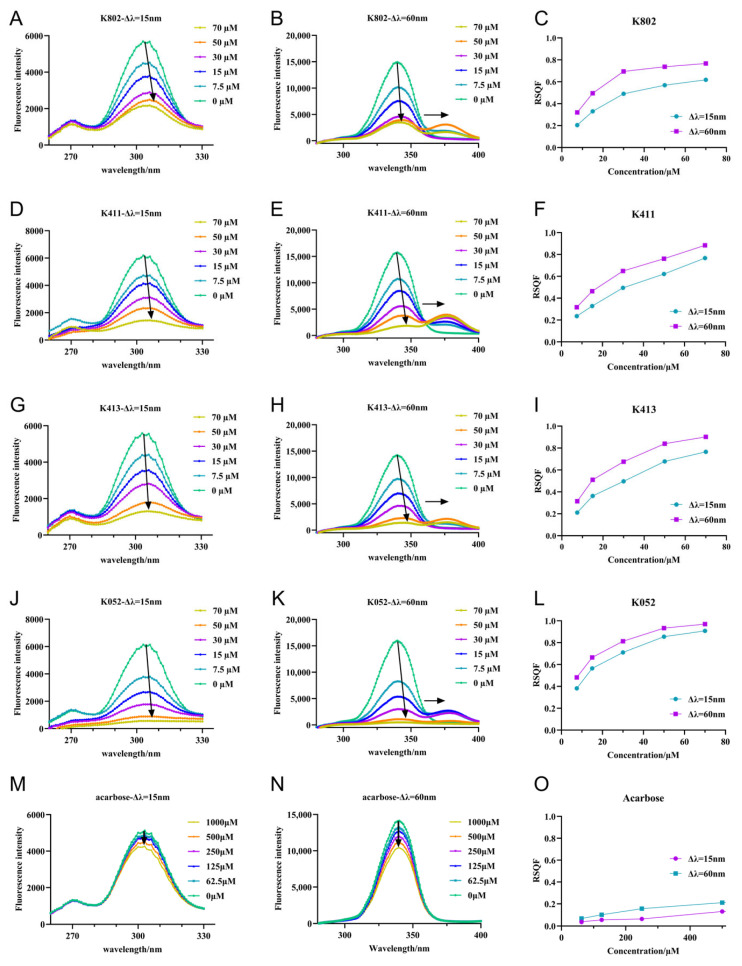
Synchronous fluorescence spectra of inhibitors and α-glucosidase. (**A**,**D**,**G**,**J**,**M**) are the synchronous fluorescence spectra of α-glucosidase by compounds and acarbose with Δλ = 15 nm. The arrow in the figure indicates the direction of shift for the maximum wavelength; (**B**,**E**,**H**,**K**,**N**) are the simultaneous fluorescence spectra of α-glucosidase by compounds and acarbose, respectively, with Δλ = 60 nm.The arrow in the figure indicates the direction of shift for the maximum wavelength; (**C**,**F**,**I**,**L**,**O**) are the RSQF maps of α-glucosidase of compounds and acarbose.

Synchronized fluorescence spectroscopy can detect changes in the microenvironment of tyrosine (Tyr) and tryptophan (Trp) residues, providing insights into protein conformational changes [21]. To further detect conformational changes in the microenvironment of the protein, synchronized fluorescence spectroscopy was generated. (Figure 8) As inhibitor concentration increased, the synchrotron fluorescence intensity of α-glucosidase at Δλ = 15 nm and Δλ = 60 nm gradually decreased. This suggests that inhibitor binding exposed the chromophore to a more aqueous environment, reducing fluorescence intensity [22]. Notably, the fluorescence peaks of Tyr residues exhibited a significant red shift, indicating that inhibitor binding increased the polarity of the Tyr residue microenvironment. Similarly, the fluorescence peak of Trp residues showed a red shift only in the presence of inhibitors, suggesting an enhancement of the polarity in the Trp microenvironment. In contrast, the fluorescence peaks of Tyr and Trp residues did not shift in the presence of the positive control acarbose, indicating that acarbose binding does not alter the microenvironment of these residues [23]. Furthermore, RSQF plots were used to evaluate the contributions of Trp and Tyr residues to the fluorescence quenching effect [23]. As shown in Figure 8, the RSQF values of K052 at Δλ = 15 nm and 60 nm were nearly equal, suggesting that Trp and Tyr residues contribute similarly to the fluorescence quenching. For K802, K411, and K413, the RSQF values for Trp (Δλ = 60 nm) were significantly higher than those for Tyr (Δλ = 15 nm), indicating that Trp plays a greater role in the intrinsic fluorescence quenching, and that these compounds bind to α-glucosidase closer to the Trp than the Tyr residues [22]. Similar findings have been reported for the binding of flavonoids to α-glucosidase [24]. The very low RSQF value of acarbose further suggests that its interaction with α-glucosidase does not significantly alter the structure or microenvironment of Tyr and Trp residues.

### 2.7. Computational Prediction of Binding Modes via Molecular Docking

**Figure 9:**
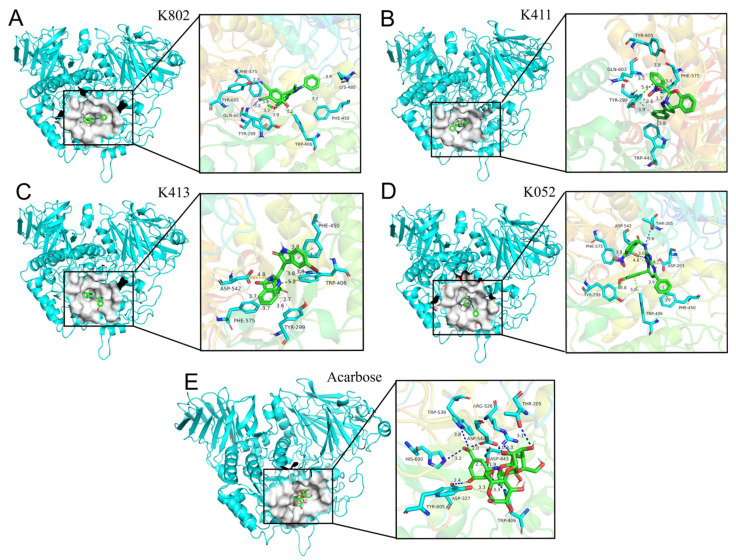
Molecular docking of α-glucosidase with compounds and acarbose. (**A**) Analysis of the interaction forces between inhibitor K802 and α-glucosidase; (**B**) Analysis of the interaction forces between inhibitor K411 and α-glucosidase; (**C**) Analysis of the interaction forces between inhibitor K413 and α-glucosidase; (**D**) Analysis of the interaction forces between inhibitor K052 and α-glucosidase; (**E**) Analysis of the interaction forces between Acarbose and α-glucosidase. The dashed blue lines represent hydrogen bonds, the dashed gray lines represent hydrophobic interactions, the dashed green lines represent π-π stacking, and the dashed yellow lines represent salt bridges.

Human α-glucosidase (commonly referred to as maltose amylase, MGAM) catalyzes the terminal glucose-releasing step in starch digestion. The active site of its N-terminal domain (NtMGAM) forms a pocket structure within a (β/α)_8_-barrel fold, primarily comprising C-terminal β-strands and featuring two critical sugar-binding subsites (−1 and +1) [25]. Molecular docking reveals that the clinical drug acarbose binds this site via multiple hydrogen bonds, particularly with its ring structure (Figure 9), occupying both the −1 and +1 subsites. Interaction analysis demonstrates that compounds K802, K411, and K413 engage the α-glucosidase active region through hydrophobic interactions, hydrogen bonds, and salt bridges with residues K802, K411, and K413. Notably, K052 forms additional stabilizing contacts: Hydrogen bonding with the acid/base catalyst Asp542, enhancing inhibitor anchoring. A salt bridge with Asp203 (located in the N-terminal domain ring, residues 200–217) upon occupying the +1 subsite, promoting tighter binding [25]. In conclusion, the inhibitors predominantly bind via hydrophobic interactions, hydrogen bonds, and salt bridges, with hydrogen bond count and specific residue interactions being decisive for binding affinity.

### 2.8. In Vitro and In Vivo Evaluation of Starch Digestion Inhibition

**Figure 10:**
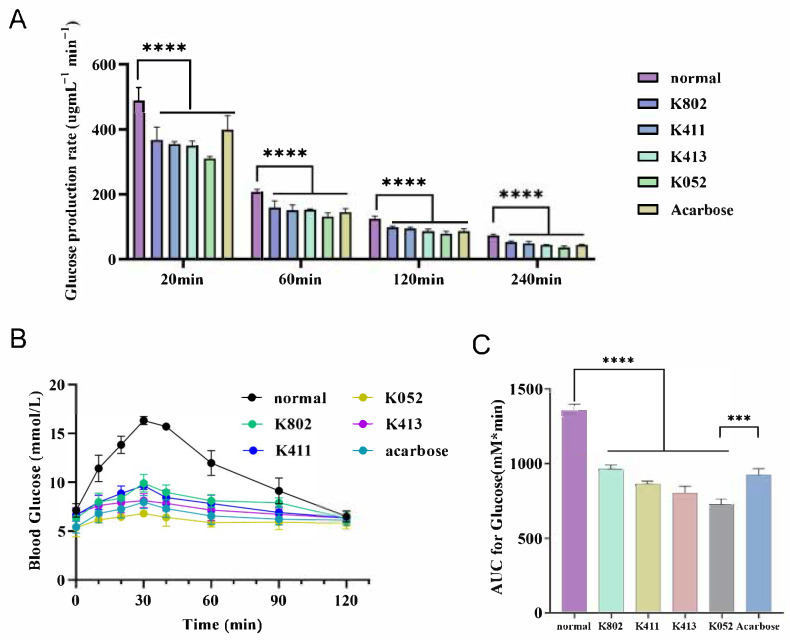
In vitro and in vivo effect of inhibitors on starch digestion and postprandial blood glucose. (**A**) In vitro inhibition rate of starch digestion in the presence or absence of inhibitors. (**B**) Blood glucose levels and (**C**) corresponding area under the curve (AUC) following intragastric administration of a starch suspension with or without inhibitors. Statistical significance is indicated as *** *p* < 0.0001 and **** *p* < 0.00001. NM, normal; Acar, acarbose.

To evaluate the molecules’ potential for modulating postprandial glucose, we assessed its inhibitory effects on starch digestion in vitro and in vivo (Figure 10). Based on established kinetics models [26], the 180 min in vitro digestion process comprises two distinct phases: a rapid phase (0–30 min) and a slow phase (30–150 min). Our extended 240 min in vitro experiments demonstrated that the inhibitor significantly reduced starch digestion rates. This effect was most pronounced during the initial rapid phase, where inhibitor-treated samples showed markedly reduced digestion compared to controls (Figure 10A). Notably, several test inhibitors exhibited efficacy comparable to acarbose in suppressing in vitro digestion.

Furthermore, pre-administration of select inhibitors to normal mice significantly attenuated the rapid blood glucose spike within 30 min prior to starch suspension ingestion (*p* < 0.01 vs. controls; Figure 10B). Key observations include the following: Multiple compounds achieved postprandial glycemic control comparable to acarbose while mitigating its gastrointestinal side effects (e.g., flatulence, diarrhea), positioning them as promising therapeutic alternatives for impaired starch digestion. Area-under-the-curve (AUC) analysis confirmed significantly reduced total glycemic exposure in the inhibitor-treated group and the acarbose-treated group versus controls (*p* < 0.001), demonstrating effective suppression of postprandial hyperglycemia. The inhibitors uniquely modulated starch digestion kinetics, not only reducing glucose spikes but also preventing pathological starch dumping (malabsorption-triggered microbial fermentation). This dual-action profile suggests clinical utility for obesity/diabetes management where conventional α-glucosidase inhibitors are poorly tolerated.

## 3. Materials and Methods

### 3.1. Materials and Reagents

α-Glucosidase isolated from Saccharomyces cerevisiae (EC 232-604-7, lyophilized powder, ≥100 U/mg protein) and α-amylase isolated from porcine pancreas (EC 232-556-6, powder, ≥5 U/mg solid) were purchased from Sigma-Aldrich Co. (St. Louis, MI, USA). The inhibitors used in this study were purchased from the ChemDiv website. Unless otherwise stated, all other reagents used in this experiment were analytically pure reagents purchased from Sigma-Aldrich Co. (St. Louis, MI, USA).

### 3.2. Machine Learning Screening

Summarize the 151 α-glucosidase inhibitors reported to date and combine them with 149 other ineffective compounds to form a dataset of 300 compounds for modeling. (Supplementary Table S4). In training, we labeled active compounds as 1 and inactive compounds as 0. The whole dataset was randomly divided into 80% as the training set and 20% as the test set. The RDKit (208), Drug tax (163), and ECFP MACCS (1191) features of 300 compound datasets totaling 1562 molecular descriptors were extracted as independent variables for this screening. After data organization and dimensionality reduction, eight ML methods, including decision tree (DT), k-nearest neighbors (kNN), support vector machine (SVM), random forest (RF), extremely randomized trees (ERT), adaptive boosting (AdaBoost), gradient boosted trees (GBT), and eXtreme gradient boosting (XGBoost), were utilized to build training set-based classification models. The parameters of each classification algorithm are optimized by a grid search algorithm that exhaustively considers all combinations of parameters. All classification algorithms were executed in the open source ML toolkit scikit-learn (version 0.18) for Python (https://www.python.org/). During the variable selection process, unnecessary variables are removed using the SelectKbest feature selection algorithm [27]. Parameter estimation of the eight ML methods was performed using the GridSearchCV module in the sklearn standard library. Using the TOPSIS algorithm, a comprehensive evaluation of seven metrics—Accuracy, Sensitivity, Kappa, MCC, F1 score, Precision, and Specificity—was conducted to select the optimal learning model. This model was then applied to screen over 100,000 compounds in the ChemDiv database, ultimately forming an initial compound library.

### 3.3. Virtual Screening

The initial screened compound library was further screened using the VS technique. In this experiment, AutoDock Vina software Version 1.2.2 was used for VS [28]. In the first step, the desired protein crystal structures were determined, and 2QMJ and 1XCW were selected as the crystal structures of α-glucosidase and α-amylase from the protein database, followed by water molecule removal and hydrogenation of the protein crystal structures. The second step was to prepare the small molecule structures. The 3D structures of the inhibitors were drawn using ChemBiodraw Ultra 14.0 to minimize the energy of the small molecules. The third step is to generate a docking box for the center and perform docking screening using AutoDock Vina. The center coordinates of the α-glucosidase docking box are (X = −20.431, y = −7.475, z = −8.691), with dimensions set to 22.5 × 22.5 × 22.5. The center coordinates of the α-amylase docking box are (X = 10.481, y = 15.523, z = 50.003), with dimensions set to 22.5 × 22.5 × 22.5. This process starts with docking the primary screened compounds with α-glucosidase, and compounds with a docking score < −8.5 are selected as the second screened compounds. Subsequently, the second screened compounds were again docked with α-amylase, and this time compounds with a docking score > −5.0 were selected as the final candidate compounds. The threshold selection is based on our empirical choice, aiming to balance screening efficiency and the number of compounds for subsequent experiments. The structures of the obtained candidate compounds were clustered and analyzed to obtain representative compound species for activity analysis.

### 3.4. In Vitro α-Glucosidase Activity Assay

The in vitro α-glucosidase activity assay was determined spectrophotometrically as previously described with only minor modifications [29]. A phosphate-buffer solution at pH 6.8 was configured to obtain 0.5 U/mL of α-glucosidase and 0.6 mM of p-nitrophenyl-diglucoside (pNαGP). All test chemicals, including the standard drug acarbose, were dissolved in DMSO to form a 10 mM master mix. Gradient dilution with phosphate-buffer solution was performed to obtain sample solutions of different concentrations. First, different concentrations of compounds (10 µL), enzyme solution (40 µL) and potassium phosphate buffer (100 µL) were pre-incubated in a 96-well plate for 10 min at 37 °C. Then, 50 µL of substrate (pNαGP, 0.6 mM) was added to each microtiter well and incubated for 20 min at 37 °C, and the absorbance at 405 nm was measured to detect the change in enzyme activity. Acarbose and DMSO were used as standard and control inhibitors, respectively.

The enzyme inhibitory activity of the tested compounds was calculated as follows:

$$\alpha -glucosidase\;inhibition\;rate\left(\%\right)=\frac{Abs\;control-Abs\;sample}{Abs\;control}\times 100$$

The IC_50_ values of the tested compounds were calculated using nonlinear fitting (logit method).

### 3.5. In Vitro α-Amylase Activity Assay

The in vitro α-amylase activity assay was determined spectrophotometrically as previously described with only minor modifications [15]. A phosphate-buffer solution at pH 6.8 was configured to obtain α-amylase 5 U/mL and 2-chloro-4-nitrophenyl α-D-maltotrioside (G3-CNP) 0.6 mM. All test chemicals, including the standard drug, acarbose, were dissolved in DMSO to form a 10 mM master mix. Gradient dilution with phosphate-buffer solution was performed to obtain sample solutions of different concentrations. First, different concentrations of compounds (10 µL), enzyme solution (40 µL) and potassium phosphate buffer (100 µL) were pre-incubated in a 96-well plate at 37 °C for 10 min, then 50 µL of substrate (G3-CNP, 0.6 mM) was added to each microtiter well and incubated at 37 °C for 20 min, and the absorbance was measured at 405 nm to detect the change in enzyme activity. Acarbose and DMSO were used as standard and control inhibitors, respectively.

$$\alpha -amylase\;inhibition\;rate\left(\%\right)=\frac{Abs\;control-Abs\;sample}{Abs\;control}\times 100$$

The IC_50_ values of the tested compounds were calculated using nonlinear fitting (logit method).

### 3.6. Analysis of Inhibition Kinetics

To further investigate the inhibition kinetics of the candidate compounds, α-glucosidase activity was determined at different substrate concentrations [29]. The Lineweaver-Burk expression method in Equation (1) was used to test the reaction of a series of compounds with the substrate pNαGP, and the values of maximum velocity (*V_max_*) and Michaelis-Menten constant (*K_m_*) were calculated [15].

(1) $$\frac{1}{v}=\frac{K_{m}}{V_{max}}\frac{1}{\left[S\right]}+\frac{1}{V_{max}}$$

where *V_max_* and *K_m_* are the maximum velocity of the enzyme and the Michaelis-Menten constant in the absence of inhibitor, respectively. [S] is the concentration of substrate and inhibitor.

(2) $$Slope=\frac{K_{m}}{V_{max}}+\frac{K_{m}}{V_{max}\cdot K_{i}}\left[I\right]$$

where *v* is the initial reaction rate, *V_max_* is the maximum initial reaction rate, [*I*] is the concentration of corrosion inhibitor, and *K_m_* is the Michaelis-Menten constant.

### 3.7. Fluorescence Quenching Experiment

The fluorescence quenching experiment was modified somewhat based on previous studies [30,31]. Fluorescence was determined by titrating α-glucosidase (1.0 mL, 2 U/mL) with different concentrations of inhibited solutions (1.0 mL, 0–200 mM) and left to equilibrate for 5 min. The fluorescence intensity of the reaction solution was measured in a quartz cuvette using a fluorescence spectrometer (F-7100, Hitachi, Tokyo, Japan) at different temperatures (305.15, 310.15 and 315.15 K) (1.0 cm path length). The emission wavelength was set at 295~500 nm, the excitation wavelength at 280 nm, and the excitation and emission slit widths were set at 5 nm. Fluorescence burst analysis was performed based on the Stern-Volmer (3) equation.

(3) $$\frac{F0}{F}=1+Kq\tau 0\left[Q\right]=1+Ksv\left[Q\right]\;$$

*F* and *F*_0_ are the fluorescence intensities with and without inhibitor, respectively, *K_sv_* is the Stern-Volmer bursting constant, *K_q_* is the bimolecular bursting constant, and [*Q*] is the concentration of inhibitor.

When the bursting mode conforms to static bursting, the binding constant *K_a_* and the number of binding sites for each enzyme can be calculated from Equation (4).

(4) $$log\frac{F0-F}{F}=logKa+nlog\left[Q\right]\;$$

*K_a_* is the binding constant and n is the number of binding sites.

The thermodynamic parameters and types of forces between the enzyme and the inhibitor were analyzed using the Vant ‘t Hoff Equation (5).

(5) $$\log K_{a}=-\frac{∆H}{2.303RT}+\frac{∆S}{2.303R}$$

(6) ∆G=∆H−T∆S

*K_a_* is the binding constant, R is the universal gas constant, and T is the test temperature (300.15 K, 305.15 K, 310.15 K). The thermodynamic parameters ∆*H*, ∆*S*, and ∆*G* denote the change in heat, entropy, and free energy, respectively.

Changes in the microenvironment of tyrosine (Tyr) and tryptophan (Trp) residue fluorophores were detected simultaneously [22]. The excitation and emission wavelength intervals were set to 15 nm and 60 nm, respectively, and the synchronized fluorescence spectra from 260 to 320 nm were determined. The synchronized fluorescence burst ratio (RSFQ) was calculated as follows:

(7) $$RSQF=1-\frac{F}{F_{0}}\;$$

### 3.8. Molecular Docking

This experiment uses AutoDock Vina for molecular docking [28]. The first step was to prepare the protein crystal structure. 2QMJ was selected from the protein database as the crystal structure for α-glucosidase docking, and the protein crystal structure was processed by removing water molecules and adding hydrogen atoms. The second step was small molecule preparation. ChemBiodraw Ultra 14.0 (Perkin Elmer Instruments Co., Waltham, MA, USA) was used to draw the 3D structure of the inhibitor and minimize the energy of the small molecule. In the third step, docking cassettes were generated for the centers and docked using AutoDock Vina. Ten poses were generated during docking, and the voxel with the highest Glide score was selected to study the interaction between the inhibitor and α-glucosidase.

### 3.9. In Vitro Starch Digestion Inhibition Assay

The effect of inhibitors on in vitro starch digestibility was determined using a slightly modified Englyst method [32,33]. Cornstarch (300 mg) and guar gum (25 mg) were added to a 50 mL centrifuge tube and dissolved in 7.5 mL of distilled water. The tubes were boiled in a boiling water bath for 10 min, cooled to room temperature, and sodium acetate buffer (2.5 mL, 0.4 M, pH 5.2, containing 0.18% (*w*/*v*) CaCl_2_) was added. After equilibrating the sample tubes at 37 °C for 15 min, fresh porcine trypsin extract, amyloglucosidase, and inhibitor mixture (5.5 mL) were added to hydrolyze the starch. Meanwhile, the no-inhibitor group and the acarbose group were used as blank and positive controls. At 20 min, 60 min, 120 min, and 240 min, 250 μL of starch hydrolysate was taken from the centrifuge tube, and 10.0 mL of 66% (*v*/*v*) ethanol was added. Gluconeogenesis was measured using a D-glucose assay kit (GOPOD).

### 3.10. In Vivo Starch Digestion Inhibition Experiments

After the in vitro validation, in order to further characterize the effect of the inhibitor on postprandial glycemic control, we performed in vivo characterization by gavage in mice [26]. Eight-week-old male C57BL/6J mice (purchased from Beijing Huafukang Laboratory Animals, Beijing, China) were used for the experiments. The animal experimental procedures were approved by the Ethics Committee on Laboratory Animal Management and Welfare of Beijing Huafuqian Times Technology Co. Ltd. (Ethical Review Approval No. HYSD2023-04), and local and national ethical guidelines were strictly observed. All mice were placed in a controlled environment with a temperature of 25 °C, relative humidity of 60%, and a light/dark cycle of 12/12 h. Following overnight fasting, gastric intubation was performed on mice in the control group, acarbose group, K802 group, K411 group, K413 group, and K052 group. The control group received saline infusion to eliminate confounding factors during administration. The acarbose group received a 10 mg/kg acarbose solution as a positive control. The remaining groups each received a corresponding concentration (10 mg/kg) of the test compound dissolved in saline solution to evaluate inhibitor efficacy. 2 g/kg of starch was given after 5 min, and the test compounds were administered at 0 min, 10 min, 20 min, 30 min, 40 min, 60 min, 90 min and 120 min. Blood was collected from the tail vein at 0 min, 10 min, 20 min, 30 min, 40 min, 60 min, 90 min, and 120 min time points to determine the blood glucose level, and the trapezoidal rule was used to calculate the area under the curve (AUC).

### 3.11. Data Processing

Unless otherwise stated, the experiments were replicated three times, and the data are expressed as the mean ± standard deviation of the three replications. The figures were created using GraphPad Prism 9.0 (GraphPad, La Jolla, CA, USA) and Qrigin 8.0 (Origin Lab Inc., Northampton, MA, USA). One-way analysis of variance (ANOVA) was performed using GraphPad Prism 9.0 (GraphPad, La Jolla, CA, USA), and statistical significance was analyzed at the 95% confidence level.

## 4. Conclusions

In this study, we demonstrate that integrating VS with ML (XGBoost mode) is an efficient strategy for developing selective α-glucosidase inhibitors. Four validated compounds showed significantly higher inhibitory activity and selectivity against α-glucosidase compared to the reference drug acarbose. Fluorescence quenching assays revealed a notably high affinity of these molecules for α-glucosidase, with their interaction altering the enzyme’s molecular structure and increasing the microenvironmental polarity around the Tyr and Trp residues. Molecular docking further suggested the important binding positions of molecules to the protein. Additionally, all four compounds enhanced binding within the active site by forming hydrogen bonds or salt bridges with Asp443 and Asp526. The combined effects of these mechanisms effectively inhibit substrate binding, thereby strengthening the inhibitory effect. This has been validated through in vitro and in vivo starch digestion experiments. Further exploration of the intestinal action mechanism and optimal dosing regimens for these selective α-glucosidase inhibitors is needed to fully harness their glycemic control potential.

## Figures and Tables

**Table 1:** IC_50_ values of four candidate compounds and the reference drug against two starch-digesting enzymes.

Number	α-Glucosidase IC_50_ (μM)	α-Amylase IC_50_ (μM)
K802	74.67 ± 4.10	>500
K411	67.85 ± 2.18	>500
K413	66.30 ± 7.17	>500
K052	23.03 ± 3.27	>500
Acarbose	562.22 ± 20.74	28.23 ± 2.34

## Data Availability

Data will be made available on request.

## Supplementary Materials

The following supporting information can be downloaded at: https://www.mdpi.com/article/10.3390/molecules30193996/s1, Table S1. Candidate compounds; Table S2. Inhibition kinetics parameter; Table S3. Quenching constants (K_sv_), binding constants (K_a_), and number of binding sites (n) of inhibitors with α-glucosidase interaction at different temperatures; Table S4: A dataset of 300 compounds for modeling; Table S5: A dataset of 300 compounds for modeling-2; Table S6: Identified 54,239 active compounds.
