# Supplementary material for: Identification of Selective α-Glucosidase Inhibitors via Virtual Screening with Machine Learning
Source: Molecules. 2025 Oct 6;30(19):3996. doi: 10.3390/molecules30193996 (PMC12525602; doi:10.3390/molecules30193996)
Supplement: Supplementary file 1 [file molecules-30-03996-s001.zip › molecules-3867159-supplementary.pdf]

Table S1 Candidate compounds

Ligand name	Smiles
8011-9275	c1ccc2C(C3=C(c2c1)NC1=C(C(c2ccccc12)=O)C31C(Nc2ccccc12)=O)=O
D733-0563	CC(N1CCC2(CC1)N1C(CC(c3ccc(C)cc3)=N1)c1ccccc1O2)=O
V021-8242	Cc1cccc(c1)C(N1C(COC12CCN(CC2)C(C(C)(C)C)=O)C(NCc1ccccn1)=O)=O
K227-0521	c1(/c(=N/c2ccc(C(=O)N)cc2)/oc2c(c1)c(cnc2C)CO)C(=O)Nc1cc(C(F)(F)F)ccc1
5849-2324	C(c1cc2c(cc1C#N)Oc1ccccc1c1nnc(c3ccccc3)n12)#N
Y600-2167	Cc1cc(C)nc(NC(\NC2Cc3ccccc3C2)=N/C(c2ccc(cc2)F)=O)n1
4111-0003	C1C2c3ccccc3OC(c3ccccc3[N+]([O-])=O)N2N=C1c1ccccc1
K227-0782	CCOc1ccccc1/N=C1/C(=Cc2c(CO)cnc(C)c2O1)C(Nc1ccc(c(c1)F)F)=O
1319-0058	COc1ccc2c(C=C(/C(=N/c3cccc(C#N)c3)O2)C(Nc2ccccc2)=O)c1
K227-0296	Cc1ccc(cc1)NC(C1=Cc2c(CO)cnc(C)c2OC/1=N/c1ccc(C)c(c1)F)=O
2360-0346	N1(C(=O)[C@H]2[C@@]3(c4c([C@H]([C@@H]2C1=O)c1c3cccc1)cccc4)C)c1c c(C(=O)O)ccc1
2360-0433	CC(c1cccc(c1)N1C(C2C(C1=O)C1(C)c3ccccc3C2(C)c2ccccc12)=O)=O
8006-4101	CC12C3C(C(N(C3=O)c3cccc(c3)[N+]([O-])=O)=O)C(C)(c3ccccc13)c1ccccc12
8009-0366	CC(C12C3C(C(c4ccccc14)c1ccccc12)C(N(C3=O)c1cccc(c1)C(F)(F)F)=O)=O
D413-0067	COc1ccc(cc1)C1C=C(c2ccccc2)Nc2nc(NC(c3cccc(c3)[N+]([O-])=O)=O)nn12
G932-0832	CCN1CCN(CC1)c1nc(c2cnn(c3ccccc3)c2n1)Nc1ccc2c(c1)OCO2
8009-7456	CC(C12C3C(C(c4ccccc14)c1ccccc12)C(N(C3=O)c1ccccc1C#N)=O)=O
Y500-9419	CC1Cc2ccccc2N1C(c1cnn2c(cc(c3ccc(cc3)OC)nc12)C(F)F)=O
S952-0156	CCc1cccc(c1)NC(N1CC(C(N)=O)C2(CCN(CC2)c2ccc(C#N)c(c2)C(F)(F)F)C1)= O
S952-5475	C1CCN(C1)C(C1CN(CC12CCN(CC2)C1CCOCC1)c1ccc(C#N)c(c1)C(F)(F)F)=O
Z274-0575	C1(=NC2(N[C@H](C1)c1ccc(cc1)F)CCN(C(=O)C)CC2)c1c(ccc(c1)Cl)O
2523-0155	C[C@@]1(C2(C(C)O)C3=CC=CC=C3[C@H]([C@@]1(C4=O)C)C5=C2C=CC= C5)C(N4C6=CC=CC(Br)=C6)=O
6058-0088	n1(c(nc2c(c1=O)cccc2)/C=C\1/C(=O)Nc2c1cc(cc2)Br)c1ccc(C(=O)O)cc1
8007-8043	CC1(C)CC2=C(C(C3=C(CC(C)(C)CC3=O)O2)c2ccc3c(c2)OCO3)C(C1)=O
8020-8172	Cc1c(c2cc(C#N)c(C#N)cc2O)c(c2ccc(cc2)OC)nn1c1ccccc1
K227-0235	c1(/c(=N/c2cc(c(cc2)OC)OC)/oc2c(c1)c(cnc2C)CO)C(=O)Nc1cc(ccc1)C
Y021-2057	CCCC1=CC(NC(NC(/Nc2ccc3c(c2)OCCO3)=N/C(c2ccccc2)=O)=N1)=O
1657-0130	CC(C12C3C(C(c4ccccc14)c1ccccc12)C(N(C3=O)c1ccc(C)c(c1)[Cl])=O)=O
5639-0187	COC1=CC=C(O/C(C(C(NC2=CC=CC=C2)=O)=C3)=N\C4=CC=CC(C#N)=C4)C 3=C1
N023-0005	[H][C@@]12CCC3(C)C(CCC3([C@]2([H])CCC2(C[C@@H](CCC12/C=N/Nc1c cccc1)O)O)O)C1COC(C=1)=O
Z274-0609	CC(N1CCC2(CC1)NC(CC(c1ccccc1O)=N2)c1ccccc1OC)=O

Table S2 Inhibition kinetics parameter

Inhibitor	[*I*] (μM)	Type of inhibition	V_{max} (×10^{-1}μM/min)	K_m (μM)	K_{ic} (μM)
-	0	-	8.55±0.02	21.38±0.12	62.63±12.68
K802	70	Competitive	9.48±0.02	19.06±1.23	

	50		9.62±0.05	17.02±0.93	
	30		9.35±0.03	14.89±0.68	
	15		9.83±0.06	12.71±1.37	
	7.5		9.17±0.04	9.88±1.57	
	70		8.43±0.04	20.28±1.59	
	50		8.81±0.03	19.22±1.38	
K411	30	Competitive	8.62±0.04	15.35±1.05	61.28±14.38
	15		8.85±0.02	13.58±1.57	
	7.5		8.94±0.06	11.58±0.51	
	70		10.50±0.03	26.70±1.83	
	50		10.84±0.07	23.52±1.07	
K413	30	Competitive	10.92±0.06	19.70±0.32	59.69±10.61
	15		11.35±0.08	17.98±0.62	
	7.5		11.41±0.04	14.88±0.39	
	70		2.44±0.02	9.12±0.12	
	50		2.50±0.02	8.04±1.23	
K052	30	Competitive	2.47±0.05	6.10±0.99	21.56±6.38
	15		2.36±0.03	3.77±0.68	
	7.5		2.34±0.06	2.88±1.77	
	2000		2.42±0.06	1.81±4.81	
	1000		5.90±0.03	18.84±2.95	
Acarbose	500	Mixed	6.18±0.04	13.32±3.28	556.17±19.16
	250		5.92±0.06	9.58±2.29	
	125		5.96±0.06	7.47±0.95	

Note: *Vmax* is the maximum initial reaction rate, [*I*] is the concentration of corrosion inhibitor, *Km* is the Michaelis-Menten constant, and *Kic* is the competitive inhibition constant.

Table S3 Quenching constants (K_{sv}), binding constants (K_a), and number of binding sites (n) of inhibitors with α-glucosidase interaction at different temperatures.

Inhibitor	T (K)	K_{sv} (10^4 /M)	K_q $(M \cdot S)^{-1}$	R^2	n	K_a (10^5 /M)	R^2
	300.15	0.59 ±0.02	0.59×10^{12}	0.99	1.16	3.99 ±0.33	0.99
K802	305.15	0.42 ±0.04	0.42×10^{12}	0.99	0.99	0.51±0.37	0.99
	310.15	0.39 ±0.02	0.39×10^{12}	0.98	0.79	0.42±0.25	0.99
	300.15	10.50 ±0.01	11.46×10^{12}	0.99	1.15	5.05 ±0.62	0.99
K411	305.15	7.67 ±0.01	7.67×10^{12}	0.99	1.17	4.82 ±0.01	0.99
	310.15	5.51 ±0.01	5.51×10^{12}	0.99	1.15	2.56±0.01	0.99
	300.15	11.46 ±0.01	10.50×10^{12}	0.99	1.17	6.30±1.23	0.99
K413	305.15	7.99 ±0.01	7.99×10^{12}	0.99	1.20	6.18±0.01	0.99
	310.15	6.87 ±0.01	6.87×10^{12}	0.99	1.14	2.54±0.01	0.99
	300.15	41.18 ±0.01	41.18×10^{12}	0.99	1.42	78.52 ±0.01	0.99
K052	305.15	32.91±0.01	32.91×10^{12}	0.99	1.20	46.45±0.24	0.99
	310.15	27.00±0.01	27.00×10^{12}	0.99	1.02	36.72±0.06	0.99
Acarbose	300.15	0.01 ±0.01	0.01×10^{12}	0.99	1.04	0.08 ±1.23	0.98
	305.15	0.02 ±0.01	0.02×10^{12}	0.99	0.98	0.04 ±0.24	0.99

	310.15	0.03 ±0.01	0.03×10^{12}	0.99	0.98	0.02 ±0.06	0.99
